# The diverse gating mechanisms of bacterial MscS-like ion channels

**DOI:** 10.1042/BST20261105

**Published:** 2026-09-18

**Authors:** Giorgos Hiotis, Yoshitaka Nakayama, Swati Yadav, Thomas Walz

**Affiliations:** 1 Laboratory of Molecular Electron Microscopy, The Rockefeller University; New York, NY, U.S.A.; 2 Tri-Institutional PhD Program in Chemical Biology, The Rockefeller University; New York, NY, U.S.A.

**Keywords:** Bacterial ion channels, Channel gating, Mechanosensitive channels

## Abstract

Bacterial mechanosensitive (MS) channels protect cells against hypoosmotic shock by opening, in response to increased membrane tension, a transmembrane pore that allows rapid efflux of osmolytes. The MS channel of small conductance (MscS) in *Escherichia coli* has been extensively characterized both functionally and structurally, establishing its channel properties and revealing its architecture and the conformational changes that underlie channel gating. In particular, several lipids associated with MscS have been proposed to play a role in its mechanosensitivity. MscS is the founding member of the superfamily of MscS-like channels that share a structurally conserved core with MscS but feature additional structural elements that result in distinct functional properties. In addition to MscS, *E. coli* expresses five MscS-like channels. In the present review, we summarize recent advances in the structural characterization of *E. coli* MscS-like channels that revealed diverse gating mechanisms. Unlike the mechanosensation of MscS that appears to depend on its associated lipids, the mechanosensation of MscS-like channels seems to be the result of the deformation they cause on their surrounding membrane. Despite the diversity in observed gating mechanisms, we highlight unifying principles that dictate the properties of MscS-like channels and MS channels in general.

Mechanosensitive (MS) channels convert mechanical stimuli into transmembrane (TM) ion currents. Mechanosensation underlies several physiological processes in eukaryotes, such as hearing, touch, and somatosensation [1–3]. In bacteria, MS channels are primarily responsible for protection against hypoosmotic shock, serving as emergency valves that release osmolytes to reduce water influx and prevent cell lysis [4]. The first two MS channels were discovered in bacteria: the MS channels of large and small conductance (MscL and MscS, respectively) [5,6]. Patch-clamp electrophysiology on proteoliposomes revealed that activation of these channels does not require accessory proteins [5–7], leading to the ‘force-from-lipid’ principle, which states that MS channels activated by this principle react to changes in the TM pressure profile [8,9].

*Escherichia coli* MscS (*Ec*MscS) (Figure 1) is the founding member of a diverse superfamily of MscS-like channels, which have a conserved core structure but feature additional structural elements that define their functional properties [10,11]. *Ec*MscS has been studied for the last two decades, but studies of MscS-like channels have only recently gained traction (Figure 2). While MscS-like channels are also expressed in some eukaryotic organisms [12–15], the present review focuses only on bacterial MscS-like channels.

**Figure 1:**
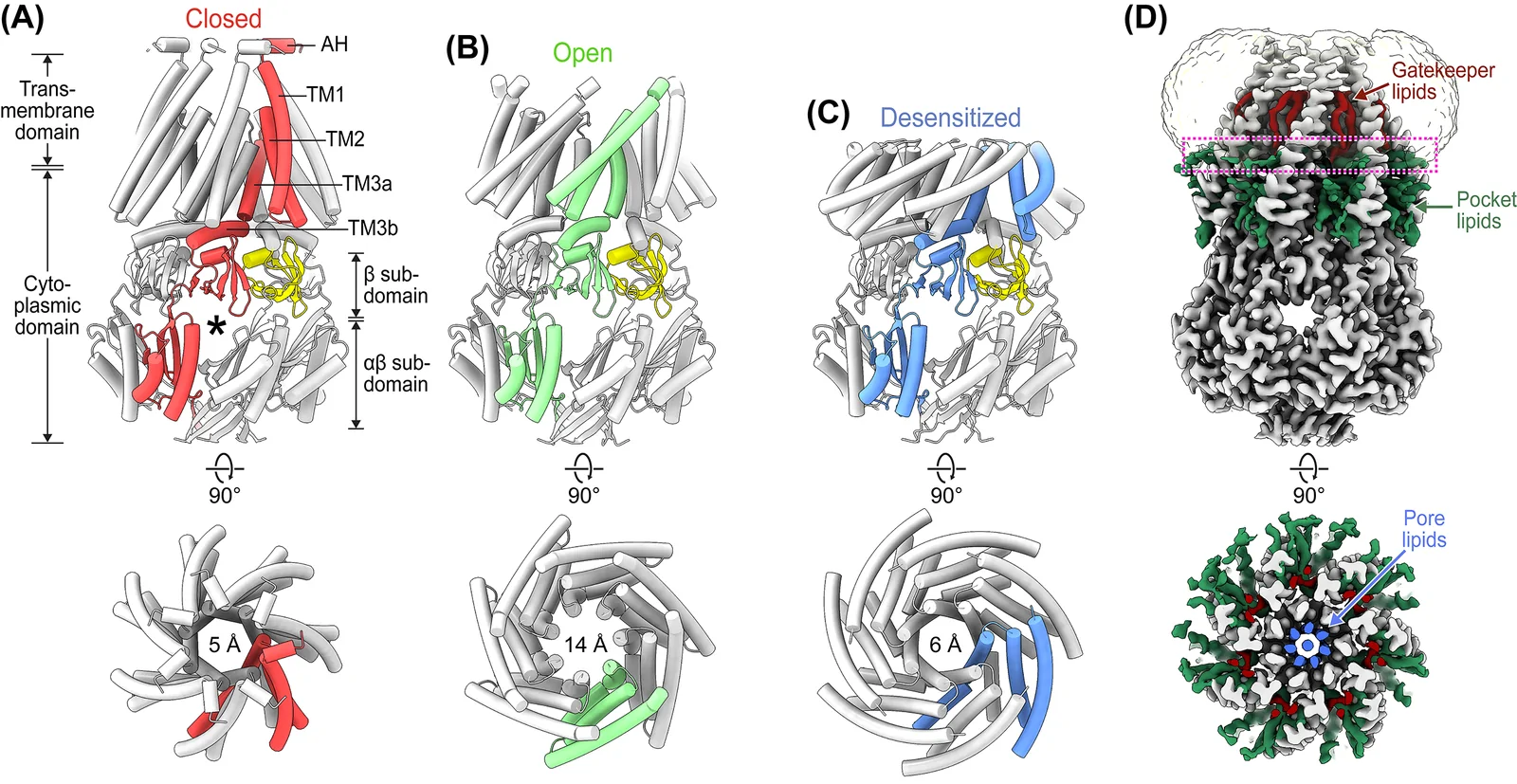
Architecture and different conformations of *Ec*MscS (**A–C**) Structures of *Ec*MscS in the closed conformation (PBD: 6VYK; one subunit highlighted in red and a neighboring β subdomain in yellow) (panel A), in the open conformation (PDB: 2VV5; one subunit highlighted in green and a neighboring β subdomain in yellow) (panel B), and in the desensitized conformation (PDB: 6VYM; one subunit highlighted in blue and a neighboring β subdomain in yellow) (panel C). *Ec*MscS consists of an amphipathic helix (AH), three TM helices, and a cytoplasmic cage that is composed of the β and αβ subdomains. The asterisk indicates a cytoplasmic fenestration. Views are parallel (top) and perpendicular to the membrane (bottom). TM helices 1 and 2 form a sensor paddle. In the closed conformation, the TM1-2 paddle of one protomer is aligned with the β subdomain of a neighboring protomer, establishing a domain-swapped configuration. In the open conformation, the TM1-2 paddle is tilted and rotated and is aligned with the β subdomain of the same protomer, resulting in a domain-aligned configuration. In the desensitized conformation, the TM1-2 paddle is even more tilted but rotated back, thus re-establishing the domain-swapped configuration. (**D**) Cryo-EM map of *Ec*MscS in DOPC nanodiscs (EMDB: 21462). The density for *Ec*MscS is shown as a solid gray surface, and the density for the nanodisc is shown as transparent surface. Views are parallel (top) and perpendicular to the membrane (bottom). The magenta box indicates the section shown in the bottom panel. Lipids associate with *Ec*MscS in three distinct locations and play specific roles: pocket lipids (green) stabilize the hydrophobic extramembranous pockets, gatekeeper lipids (yellow) stabilize the TM1-2 paddles, and pore lipids (blue) prevent ion leakage through the pore.

**Figure 2:**
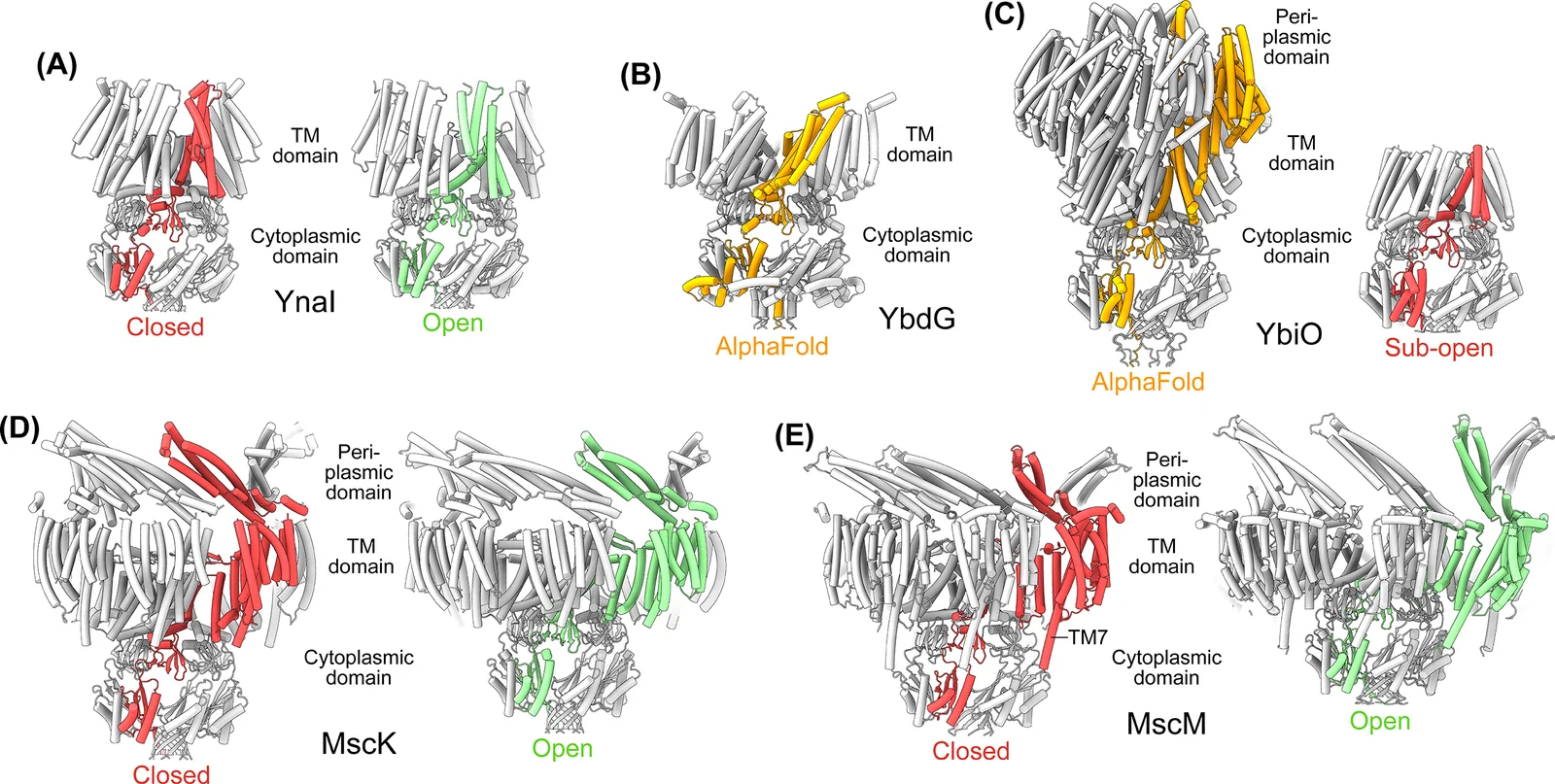
MscS-like channels expressed in *E. coli* (**A**) Cryo-EM structures of YnaI in the closed conformation (PDB: 6URT) with one subunit highlighted in red (left panel) and in the open conformation (PDB: 9H2P) with one subunit highlighted in green (right panel). Channel gating of YnaI involves minimal conformational changes in the TM domain. (**B**) AlphaFold3-Multimer prediction of YbdG with one subunit highlighted in orange [29]. (**C**) AlphaFold3-Multimer prediction of YbiO with one subunit highlighted in orange (left panel) [29], and cryo-EM structure of the MscS-like core of YbiO in a sub-open conformation (PBD: 9GO3) with one subunit highlighted in red (right panel). (**D**) Cryo-EM structure of MscK in the closed conformation (PDB: 7UW5) with one subunit highlighted in red (left panel) and in the open conformation (PDB: 7UX1) with one subunit highlighted in green (right panel). The periplasmic domain of MscK assembles into a ring. Channel gating of MscK involves a dilation of the periplasmic ring and an expansion and reduction in curvature of the TM domain. (**E**) Cryo-EM structure of MscM in the closed conformation (PDB: 11SO) with one subunit highlighted in red (left panel) and in the open conformation (PDB: 11SQ) with one subunit highlighted in green (right panel). The periplasmic domain of MscM also assembles into a ring. Channel gating of MscM involves the disassembly of the periplasmic ring and the expansion and complete flattening of the TM domain. In addition, the conformational change in the TM domain is transmitted through a cytoplasmic extension of TM helix 7 to the αβ subdomain, which rotates relative to the β subdomain and opens the previously closed lateral fenestrations in the cytoplasmic cage (see Figure 3D).

## Architecture and gating mechanism of *Ec*MscS

**Figure 3:**
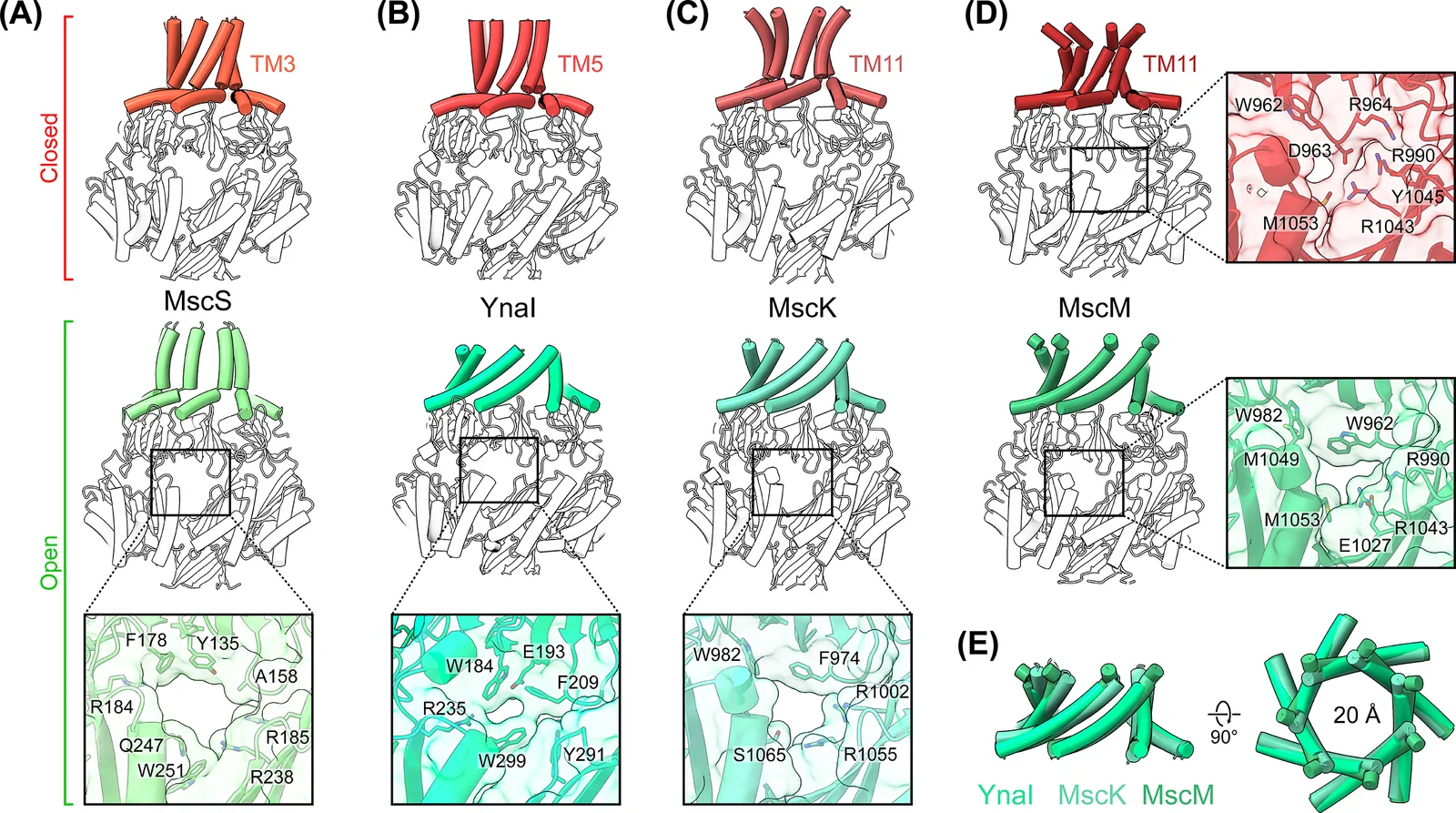
Conformation of the pore-lining helices and size of the cytoplasmic fenestrations in *Ec*MscS and MscS-like channels in the closed and open conformations (**A–D**) Structures of the pore-lining helices and cytoplasmic domains in *Ec*MscS in the closed (PDB: 6VYK) and open (PDB: 2VV5) conformations (panel A), YnaI in the closed (PDB: 6URT) and open (PDB: 9H2P) conformations (panel B), MscK in the closed (PDB: 7UW5) and open (PDB: 7UX1) conformations (panel C), and MscM in the closed (PDB: 11SO) and open (PDB: 11SQ) conformations (panel D). The pore-lining helices are highlighted in shades of red and green in the closed and open conformations, respectively. The insets show zoomed-in views of a single cytoplasmic fenestration with the fenestration-forming residues shown as sticks and labeled and the size of the fenestration visualized by the transparent surface representation of the cytoplasmic domains. In the closed conformations, the conformation of the pore-lining helices of *Ec*MscS is similar to those of MscS-like channels YnaI, MscK, and MscM. In the open conformation, the pore-lining helices of MscS orient to become more perpendicular to the membrane and move radially outward from the central axis, whereas the pore-lining helices of YnaI, MscK, and MscM lose their kink and form a continuous helix. In MscS, YnaI and MscK, the cytoplasmic domains, and thus the fenestrations, are identical in the closed and open conformations. In contrast, the cytoplasmic domain of MscM undergoes a conformational change upon channel gating, converting the fenestrations from a closed to an open state. (**E**) Superposition of the pore-lining helices of YnaI, MscK, and MscM in the open conformation. Views are parallel (left) and perpendicular to the membrane (right). The pore-lining helices of YnaI, MscK, and MscM are continuous in the open conformation, resulting in all channels having the same pore diameter of ∼20 Å, despite having different single-channel conductances. This finding suggests that the single-channel conductance of each channel is determined by the size of its cytoplasmic fenestrations rather than the diameter of its TM pore.

X-ray crystallography and cryo-EM structures revealed that *Ec*MscS forms a homoheptamer, with distinct TM and cytoplasmic domains [16–18] (Figure 1A). Each monomer contributes three TM helices. TM1 and TM2 form a membrane tension-sensing paddle, and TM3 has a kink that divides it into TM3a, which forms the pore, and TM3b, which connects to the cytoplasmic cage [16–18]. The cytoplasmic cage consists of the β and αβ subdomains, which, at their interface, form openings, termed fenestrations, that define the ion selectivity and, in some cases, the ion conduction of the channel [16–19] (Figure 3A–D). In the closed conformation, the protomers adopt a domain-swapped configuration, in which the sensor paddle of one protomer aligns with the β subdomain of the neighboring protomer [16–18].

The first structure of *Ec*MscS in an open conformation was determined by X-ray crystallography using the Ala106Val mutant [20] (Figure 1B). In this structure, the TM1-2 paddles are tilted and rotated, resulting in the protomers adopting a domain-aligned configuration, in which the sensor paddle aligns with the β subdomain of the same protomer and an open pore that allows ion conduction [20]. Later, structures of *Ec*MscS in an open-like state have been determined by cryo-EM of the channel embedded in nanodiscs formed with didecyl phosphatidylcholine and dimyristoyl phosphatidylcholine, which differ, however, in the tilt of their sensor paddles and their pore diameter [21,22]. It is currently unclear how different lipids stabilize *Ec*MscS in different open-like states. Intriguingly, the Ala106Val *Ec*MscS mutant that yielded the structure of the open conformation in a detergent environment did not reveal any density for the TM domain when the channel was reconstituted in a dioleoyl phosphatidylcholine nanodisc, raising the possibility that, in the context of a membrane, *Ec*MscS in the open state does not adopt a single defined conformation [21].

Electrophysiology has shown that the open state of *Ec*MscS is transient, and as membrane tension persists, *Ec*MscS quickly transitions to two sequential functional states [23,24]. First, *Ec*MscS desensitizes, where it no longer conducts ions but can be reactivated by increased membrane tension [23,24]. Once desensitized, *Ec*MscS will eventually transition to the inactivated state, where it can no longer be reactivated until the channel resets after several seconds in the absence of membrane tension [23,24]. A cryo-EM study using β-cyclodextrin to extract lipids from *Ec*MscS-containing nanodiscs, mimicking membrane tension, revealed a structure in which the TM1-2 paddles were more tilted than in the open state, but the protomers were in a domain-swapped configuration and the pore was closed [21] (Figure 1C). This structure was interpreted as the desensitized conformation of *Ec*MscS, but electrophysiological experiments in the same study would be more consistent with β-cyclodextrin treatment inducing an inactivated state [21]. Structures for *Ec*MscS in both the desensitized and inactivated states will be needed to unambiguously assign and understand these distinct functional states.

Molecular dynamics (MD) simulations of *Ec*MscS in the closed state revealed that the channel has extramembraneous hydrophobic pockets that are filled with lipids [25]. The volume of the pockets and number of lipids occupying them decrease when the channel transitions to the open conformation [25]. These observations led to the ‘lipids-move-first’ model as a molecular implementation of the force-from-lipids principle, in which membrane tension would pull lipids out of the pockets, thus destabilizing the closed conformation and triggering the channel to open. Subsequent cryo-EM structures of *Ec*MscS confirmed the existence of the pocket lipids, while also revealing ‘pore lipids’ in the pore of the channel and ‘gatekeeper’ (or ‘hook’) lipids in between adjacent TM1-2 paddles [17,18,21] (Figure 1D). The pore lipids are thought to prevent ion leakage in the closed state, while the gatekeeper lipids may stabilize the TM1-2 paddles and transduce membrane tension to the channel [17,21]. The lipids-move-first model has been expanded to also include the pore and gatekeeper lipids, proposing that the gatekeeper lipids are the first lipids to dissociate from *Ec*MscS and that the pore lipids diffuse out of the pore as the channel opens [21]. However, it is unclear whether pore lipids exist *in situ* or are a purification artifact and how they would egress the pore. In the desensitized state, the volume of the pockets becomes even smaller than in the open state, suggesting that further channel delipidation eventually causes desensitization [21]. Experimental support for this model came from a study that used extended incubations of *Ec*MscS with high detergent concentrations to strip lipids from the pockets, which stabilized *Ec*MscS in the open conformation [26]. Careful examination of the cryo-EM maps reveals density in the pockets consistent with several acyl chains, providing an explanation for why the detergent treatment did not induce *Ec*MscS to adopt the inactivated conformation but raising the question what exactly stabilized the channel in the open conformation.

The lipids-move-first model has limitations, as it is based on the notions that the number of lipids in the pockets dictates the conformation of the channel and that delipidation caused by membrane tension is the driving force for conformational change in *Ec*MscS. However, while it has been shown that the pocket volume and number of occupying lipids do decrease in the open and desensitized states, a recent MD study found that membrane tension alone is insufficient to strip lipids from the *Ec*MscS pockets [22]. Instead, the authors proposed that *Ec*MscS is gated by a mechanism similar to that of PIEZO1 [22,27] (see below).

## The MscS-like channels expressed in *E. coli*

*Escherichia coli* expresses *Ec*MscS and five MscS-like channels, namely YnaI, YbdG, YbiO, MscK (or KefA), and MscM (or YjeP) [28]. YnaI and YbdG have five TM helices per monomer (Figure 2A,B), whereas YbiO, MscK, and MscM have eleven TM helices (Figure 2C–E), with MscM and MscK also featuring extensive periplasmic domains [29]. Structures of YnaI, MscK, and MscM in their closed and open states revealed a diversity of gating mechanisms [30–33]. YbiO has only been structurally characterized in the closed state, and YbdG has yet to be characterized, so that their gating mechanisms remain unknown [30,34]. Curiously, YbdG does not appear to elicit ion currents when exposed to membrane tension in electrophysiology experiments, yet it can rescue *E. coli* from hypoosmotic shock, highlighting the need for further study to resolve this discrepancy [35].

YnaI is the MscS-like channel most similar to *Ec*MscS, but its functional characteristics are almost exactly opposite of those of *Ec*MscS. Unlike *Ec*MscS, which activates rapidly and then desensitizes and inactivates, YnaI has very slow activation kinetics and does not appear to desensitize/inactivate [30,31]. The single-channel conductance of YnaI is also much smaller than that of *Ec*MscS (0.1 nS versus 1.0 nS), and unlike *Ec*MscS, YnaI exhibits substantial gating hysteresis, closing at a membrane tension that is much lower than that needed for activation [30,31,36]. Cryo-EM structures of YnaI in detergent and nanodiscs revealed its conformation in the closed state [30,31] (Figure 2A). As expected, the structure of YnaI is similar to that of *Ec*MscS, with its two additional TM helices resulting in a larger paddle [30,31]. Leu154 and Met158 are the residues that form the constriction in the YnaI pore, analogous to Leu105 and Leu109 that form the vapor lock in *Ec*MscS [30,31]. Different from *Ec*MscS, which has three types of associated lipids, YnaI only has pocket lipids, which are, however, more ordered and more tightly bound than those in *Ec*MscS [30,31]. The two additional TM helices of YnaI position its core structure further out of the membrane plane compared with *Ec*MscS, making the pocket lipids of YnaI less accessible to the surrounding membrane and thus shielding them from membrane tension. Collectively, the pocket lipids in YnaI appear more structural in nature and do not play a similar role in channel gating as those in *Ec*MscS.

A recent study revealed the structure of YnaI in the open conformation, which was induced by extended incubation with a high detergent concentration, as previously done for *Ec*MscS [37] (Figure 2A). Opening of YnaI involves minimal rigid-body movements of the TM1-4 paddle and an expansion of the TM domain predominantly on the periplasmic side. The kink in TM5 disappears so that the helix becomes continuous (Figure 3B), and the diameter of the pore expands from ∼6.6 to ∼20 Å. The pocket lipids of YnaI in the open state were still clearly resolved but showed a slightly different packing. This finding is not consistent with high detergent concentrations delipidating the channel and stabilizing its open conformation, but it does support the notion that the pocket lipids are unlikely to play a role in channel gating.

MscK and MscM are the two largest MscS-like channels in *E. coli*, with a very high degree of sequence identity in TM11 and the β subdomain [33]. MscK is unusual because, in addition to membrane tension, it requires extracellular potassium for activation [38]. MscK is more mechanosensitive than *Ec*MscS and displays no noticeable gating hysteresis, whereas the mechanosensitivity of MscM is similar to that of *Ec*MscS, and the channel displays strong hysteresis [32,33,36]. MscK was structurally characterized before MscM [32] (Figure 2D). MscK features a ∼470 amino acid-long periplasmic domain predicted to be organized in two helical bundles. In the cryo-EM maps of MscK, only the membrane-proximal helical bundle was resolved, showing that it assembled into a ring structure that sits on top of a highly curved TM domain. The open conformation of the channel was obtained using the Gly924Ser mutation in the pore-lining helix of MscK (Figure 2D). Channel opening is characterized by a dilation of the periplasmic ring, a reduction in the curvature, and an increase in the footprint of the TM domain. Like in YnaI, the pore-lining helix of MscK, TM11, becomes completely continuous, and the pore diameter increases from ∼5 to ∼20 Å.

Recently, the first structures of MscM in the closed and open states were reported, revealing differences to MscK [33] (Figure 2E). In the closed state, the periplasmic ring of MscM is more dilated, and the TM domain is less curved (Figure 2E). One of the TM helices, TM7, has a long cytoplasmic extension that makes direct interaction with the αβ subdomain of the cytoplasmic cage, resulting in a distinct conformation of the cytoplasmic domain that closes its fenestrations (Figure 3D). In the open state, which was determined by purifying MscM in a buffer containing potassium chloride, suggesting that gating of MscM may depend on potassium as is the case for MscK, the periplasmic domain no longer forms a ring structure, and the TM domain becomes completely flat (Figure 2E). Deletion of the periplasmic ring was found to significantly diminish the gating hysteresis [33], suggesting that disassembly/reassembly of the ring during the gating transitions of MscM is at least partially responsible for the hysteresis seen in MscM, a notion also supported by MscK, whose periplasmic ring does not disassemble during gating and shows little hysteresis [32]. Most notably, the conformational change in the TM domain also results in a conformational change in the cytoplasmic domain, mediated by the interaction between TM7 and the αβ subdomain, which results in an opening of the fenestrations and renders the conformation of the cytoplasmic domain identical to that of MscK (Figure 3C,D). This finding established that the cytoplasmic domains of MscS and MscS-like channels serve not only as a rigid scaffold for oligomerization and a passive ion filter but can also play a role in channel gating.

Our structural understanding of YbiO is more limited. It also has eleven TM helices per monomer but a smaller periplasmic domain than MscK and MscM that is predicted to have long intrinsically disordered stretches [34]. YbiO is less mechanosensitive than *Ec*MscS and, like other MscS-like channels, displays slow activation kinetics and does not desensitize/inactivate [30,36]. A recent structure of YbiO resolved most of the three core helices TM9-11, but not TMs 1-8 [34] (Figure 2C). It may be the lack of a periplasmic ring (that rigidifies the TM domains in MscK and MscM) that causes the additional TM helices of YbiO to be highly flexible. The pore diameter of YbiO in this structure was larger than in a previous structure (∼9.5 Å versus 6.7 Å), prompting the authors to assign it as a sub-open state. The authors also performed patch-clamp electrophysiology of YbiO, finding that it can achieve a single-channel conductance of ∼3 nS while also exhibiting subconducting states of ∼0.6 nS and ∼2.1 nS. A single-channel conductance of ∼3 nS is much larger than what was measured in *E. coli* spheroplasts (∼1 nS) and would equate the single-channel conductance of YbiO to that of MscL. Further functional studies and complete structures of YbiO in the closed and open conformations will be needed to resolve current ambiguities and obtain a fuller understanding of YbiO.

## Differences and unifying principles in *E. coli* MscS and MscS-like channels

Functionally, *Ec*MscS activates very quickly upon increases in membrane tension and then desensitizes and inactivates. These characteristics differ from those of the functionally characterized MscS-like channels in *E. coli*, YnaI, YbiO, MscK, and MscM, which all show slow activation kinetics and a lack of desensitization/inactivation [30,31,33,38]. Although the degree of protection will also depend on the expression level of the channels [35,36], these characteristics suggest that *Ec*MscS responds to fast osmotic changes, whereas the MscS-like channels respond to slow ones, a notion supported by the finding that even when *Ec*MscS and *Ec*MscL are knocked out, the MscS-like channels can still protect *E. coli* from slow hypoosmotic shocks [39].

MscS and the MscS-like channels in *E. coli* with known structures in the closed and open conformations, YnaI, MscK, and MscM, all show distinct gating mechanisms. The predominant gating model for *Ec*MscS focuses on the membrane tension-induced dissociation of the gatekeeper and pocket lipids as the driving force for its conformational changes. In contrast, none of the *E. coli* MscS-like channels have gatekeeper lipids, and the pocket lipids, while present, are unlikely to play the same role as in *Ec*MscS. For YnaI, the pocket lipids are thought to play a more structural role [31,37]. For MscK and MscM, the pockets and the lipids associated with them are located within the membrane, suggesting that these also do not perform the same function as the lipids in the extramembranous pockets of *Ec*MscS [33]. Furthermore, even MscK and MscM, which appear to be similar, have unique gating features that differentiate them, in particular with the cytoplasmic cage of MscM, but not MscK, being involved in gating [32,33].

Despite these differences, the gating mechanisms of YnaI, MscK, and MscM appear to follow the model of mechanosensation proposed for the eukaryotic MS channel PIEZO1, which is based on the TM domain inducing curvature in the surrounding membrane [27,40–42]. As membrane tension increases, the deformed membrane becomes energetically unfavorable, triggering a conformational change that is characterized by a flattening and expansion of the TM domain. Therefore, the sensitivity of an MS channel would partially depend on the degree of curvature it imposes on the surrounding membrane in the resting state. In accordance with this notion, YnaI undergoes minimal changes upon opening and thus has a very high activation threshold, whereas MscK and MscM undergo substantial changes in their TM domains upon opening and are indeed more sensitive to membrane tension, consistent with previously described thermodynamic principles underlying mechanosensation [43]. The MD study mentioned above suggests that this mechanism may indeed also underlie the gating of *Ec*MscS [22].

A structural principle that unifies the MscS-like channels YnaI, MscK, and MscM but distinguishes them from *Ec*MscS is the conformational change of the pore-lining helix. In the closed state, the pore-lining helix in all these channels is kinked. However, when *Ec*MscS opens, the helix retains its kink, and the pore-forming segment, TM3a, turns parallel to the pore axis and moves radially outward, expanding the pore (Figure 3A). In contrast, when YnaI, MscK, and MscM open, their pore-lining helices lose the kink and become continuous (Figure 3B–D). The difference between the kink in the pore-lining helix either remaining in *Ec*MscS or being resolved in the MscS-like channels may provide some structural explanation for MscS-like channels, unlike *Ec*MscS, having both slow gating kinetics and gating hysteresis. Moreover, mutation of residue Gly113, which forms the kink in *Ec*MscS, to residues with higher helical propensity prevents the channel from inactivating [44], providing a potential explanation for why only *Ec*MscS but not MscS-like channels show inactivation.

Notably, while the conformation of the pore-lining helices in the MscS-like channels in the open state is identical and their pores have the same diameter (∼20 Å) (Figure 3E), the single-channel conductance of these channels is different (YnaI ∼0.1 nS, MscK ∼0.8 nS, and MscM ∼0.3 nS), showing that the pore size does not define the conductance of these channels. Instead, it is the size of the cytoplasmic fenestrations that appear to define their conductance (Figure 3A–D), but this notion awaits experimental testing.

YbdG and YbiO are structurally not yet fully characterized, and so it remains to be seen whether the principles deduced from the currently well-characterized MscS-like channels will also hold for these two channels and MscS-like channels from other bacteria.

## MscS homologs and MscS-like channels beyond *E. coli*

Our understanding of MscS homologs and MscS-like channels in bacteria other than *E. coli* is very limited. Crystal structures have been determined for MscS homologs from two other Gram-negative bacteria, *Thermoanaerobacter tengcongensis* and *Helicobacter pylori*, which show the channels in the closed conformation and reveal an architecture similar to that of *Ec*MscS [19,45].

Some Gram-negative bacteria, mostly cyanobacteria, express bacterial cyclic nucleotide-gated (bCNG) channels, which are unique MscS-like channels that feature a C-terminal cyclic nucleotide-binding domain [46,47]. Overexpression of bCNG channels did not provide protection against hypoosmotic shock in *E. coli* [46,47], suggesting that some MscS-like channels may have evolved to perform specialized roles in their host organisms other than (or in addition to) hypoosmotic shock protection, a subject that remains poorly explored. In addition to a lack of understanding of their physiological roles, there is currently also no structural data on bCNG channels that would inform on what role cyclic nucleotides may play in channel gating.

The Gram-positive bacterium *Bacillus subtilis* appears to express three MscS homologs, with their expression levels varying based on the growth phase and the salinity of the environment [48–51]. Surprisingly, *B. subtilis* remains unsusceptible to hypoosmotic shock in the stationary phase even when all known MS channels are knocked out [49]. However, the electrophysiological properties and structures of these channels remain to be determined.

*Corynebacterium glutamicum*, another Gram-positive bacterium, is important for the industrial production of glutamic acid. It expresses one MscS homolog (MscCG2) and one MscS-like channel (MscCG), which are both involved in glutamate excretion [52–55]. Electrophysiological analysis of MscCG showed that this channel functions as an MS channel, albeit with channel properties that differ from those of *Ec*MscS [55,56]. In contrast, MscCG is not required to protect *C. glutamicum* against hypoosmotic shocks, but deleting it significantly reduces glutamate excretion, suggesting that MscCG is another example of an MscS-like channel that has evolved away from providing protection against hypoosmotic shock.

## Concluding remarks

The study of MscS homologs and MscS-like channels has provided important insights into how membrane proteins can convert mechanical stimuli into TM ion currents. Structural and functional studies have revealed a remarkable diversity of channel properties and gating mechanisms but also some unifying principles. However, our current information remains very limited, and further studies are likely to unveil an even greater structural and functional diversity in this superfamily of MS channels.

## Perspectives

- Channels of the MscS-like superfamily are expressed in almost every bacterium and serve mostly as protection against hypoosmotic shocks. Studies of MscS and MscS-like channels have provided important insights into the principles underlying mechanosensation that transcend bacteria.
- Channels of the MscS-like superfamily have distinct structures and use different gating mechanisms that define their functional characteristics, allowing them to respond in a nuanced way to different types of hypoosmotic shocks.
- Little is known about channels of the MscS superfamily beyond those expressed in *E. coli*, requiring more work to establish whether principles deduced from *E. coli* MscS and MscS-like channels also apply to these channels from other bacterial and eukaryotic species. It also remains to be explored whether MscS-like channels serve functions other than protection against hypoosmotic shock.

## Open Access

Open access for this article was enabled by the participation of Rockefeller University in an all-inclusive Read & Publish agreement with Portland Press and the Biochemical Society.

## Abbreviations

bCNG: bacterial cyclic nucleotide-gated
*Ec*MscS: *Escherichia coli* MscS
MD: molecular dynamics
MS: mechanosensitive
MscS: Mechanosensitive channel of small conductance
TM: transmembrane
